# Novel Variant of the Androgen Receptor Gene in a Patient With Complete Androgen Insensitivity Syndrome and Polyorchidism

**DOI:** 10.3389/fendo.2018.00795

**Published:** 2019-01-17

**Authors:** Ilze Konrade, Julija Zavorikina, Aija Fridvalde, Dmitrijs Rots, Ieva Kalere, Ilze Strumfa, Maija Dambrova, Linda Gailite

**Affiliations:** ^1^Department of Internal Medicine, Riga Stradins University, Riga, Latvia; ^2^Department of Endocrinology, Riga East University Hospital, Riga, Latvia; ^3^Scientific Laboratory of Molecular Genetics, Riga Stradins University, Riga, Latvia; ^4^Department of Pharmacy, Riga Stradins University, Riga, Latvia; ^5^Department of Pathology, Riga Stradins University, Riga, Latvia; ^6^Department of Human Physiology and Biochemistry, Riga Stradins University, Riga, Latvia

**Keywords:** complete androgen insensitive syndrome, androgen receptor (AR) gene, polyorchidism, supernumerary testis, novel genetic variants, case

## Abstract

**Introduction:** Complete androgen insensitivity (CAIS) in 65–95% cases is caused by pathogenic allelic variants (mutations) in the gene encoding androgen receptor (*AR* gene) and is characterized by female phenotype development with a male karyotype (46, XY). Patients are usually diagnosed during puberty and undergo gonadectomy due to increased testicular germ cell tumor risk. Only a few outcomes have been reported in older individuals with postponed gonadectomy.

**Case presentation:** A 48-year-old CAIS patient presented with polyorchidism (four testes) without gonadal malignancies. Genetic testing identified a novel allelic variant in the *AR* gene [c.2141T>G (p.Phe805Cys)] causing the clinical symptoms.

**Conclusion:** We have described a unique patient with CAIS and polyorchidism without malignancies in her late 40's bearing a novel likely pathogenic variant in the *AR* gene.

## Introduction

Complete androgen insensitivity syndrome (CAIS) is a congenital disorder ofi sex development that is characterized by a female phenotype and a 46, XY karyotype due to allelic variants in the androgen receptor (*AR*) gene (NCBI Gene ID: 367, OMIM#313700), which is located in the X-chromosome (1, 2). These changes lead to complete resistance to the biological actions of androgens and fetal sex differentiation into a female phenotype. In adolescence, CAIS is characterized by excess aromatization of androgens to oestrogens and the absence of opposing androgen action due to androgen receptor dysfunction with consequent development of secondary female characteristics (2, 3). For a long time, generally accepted recommendations suggested performing gonadectomy after puberty due to the risk of malignancy (4). However, recent studies have revealed a slightly lower risk of developing testicular germ cell tumors of approximately 5% in androgen insensitivity syndrome (5), and although germ cell neoplasia *in situ* is found in 14% of patients, malignant progression appears to be a rare (6). Therefore, the practice of routine prophylactic gonadectomy in adults with CAIS appears questionable and the recommendations—less stringent (7).

## Case Summary

### Clinical Description

This study was carried out in accordance with the recommendations of “Central Ethical Committee of Latvia” with written informed consent from all subjects. All subjects gave written informed consent in accordance with the Declaration of Helsinki. For control samples there were only genotyping performed, for patient sample were obtained written informed consent for study and publication, clinical data, and anamnesis were collected retrospectively.

A 48-year-old patient was admitted to the hospital in August 2016 with a suspected testicular germ cell tumor due to CAIS. The patient's main complaints included progressive and transitory edema, general malaise and a mobile formation in the left groin gradually grown to 2.5 cm over the previous 4–5 months. General examination showed a female phenotype (Prader stage 0, Tanner grade V breast development), generalized edema (stage I/II), Body Mass Index (BMI) 32 kg/m^2^ and an elevated blood pressure of 180/120 mmHg. During physical examination, a freely moveable, non-painful oval mass was noted in the left inguinal canal. A similar smaller mass was also found in the right inguinal canal. External genital organs were normal on examination. A hypoplastic, yet functional, vagina with a vaginal canal length of 4.5 cm was present. The patient was sexually active and exhibited sexual well-ness.

CAIS had been detected in 1986, when the patient was examined due to primary amenorrhea, and her karyotype was analyzed and confirmed 46, XY karyotype. At the age of 26, the patient underwent right side hernia repair surgery during which the descended testis was mistakenly reduced to the abdominal cavity without gonadectomy.

The patient had a medical history of particular interest, which included bilateral middle cerebral artery aneurysm embolization with microspirals. After procedure there were followed without discontinuation of treatment with 5 mg of Perindopril and BP was 125/80 mmHg on this treatment.

At the time of admission to the hospital, a biochemical evaluation revealed expected luteinizing hormone (LH), follicle-stimulating hormone (FSH), testosterone, and androstenedione levels. The dynamics of all the hormonal levels are shown in Table 1.

**Table 1:** Preoperative and postoperative laboratory blood tests in the patient.

**Hormone, units**	**Normal range for males**	**Preoperatively (August 2016)**	**After the first surgery (September 2016)**	After the second surgery (December 2016)^*^
LH, IU/L	1.4–7.7	13.2	16.0	24.6
FSH, IU/L	1.5–14	15.4	22.9	40.6
Estradiol, pg/ml	< 56	48.4	46.6	21.5
Testosterone, ng/ml	3.3–8.05	18.27	5.28	0.14
Androstenedione, ng/ml	0.3–3.3	3.79	2.63	3.42

Testing was performed for germ cell malignancy markers and showed normal levels: beta-human chorionic gonadotropin < 1.20 mIU/mL (reference range < 5 mIU/mL), and alpha-fetoprotein 1.78 ng/mL (normal range < 6.7 IU/mL).

**Figure 1:**
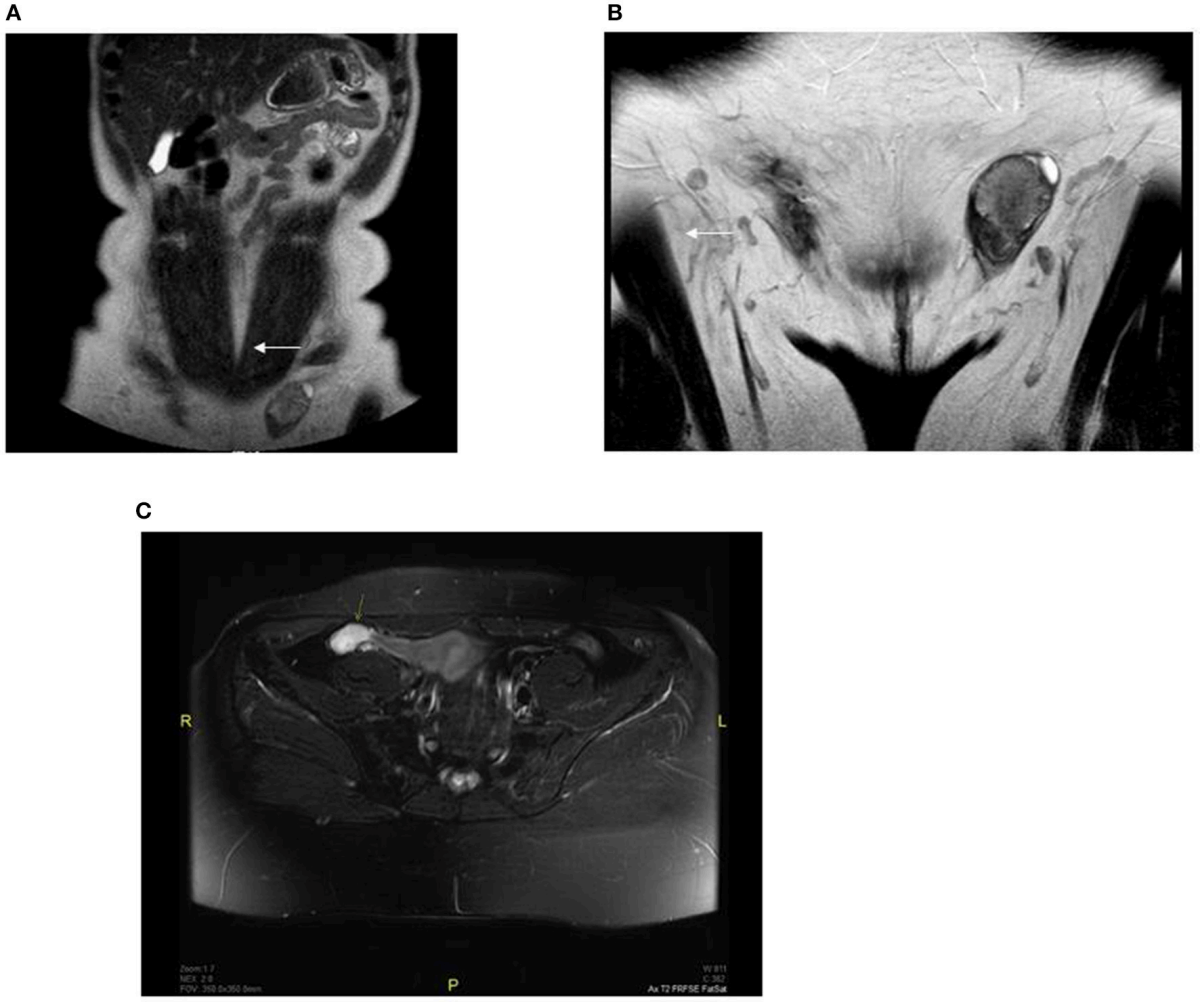
Preoperative MRI revealed oval structures without follicular activity: **(A)** right side (showed with an arrow) −1 × 1.3 cm, located near the anterior abdominal wall; **(B)** left side (showed with an arrow) −2.6 × 1.6 cm, located in the left inguinal canal; **(C)** pelvic MRI fat-suppressed T2 sequence of the right hypogastrium with a residual testicular structure 1.2 × 2.4 × 3.3 cm (showed with an arrow).

Further outpatient investigations including a pelvic ultrasound and magnetic resonance imaging (MRI) examination revealed oval structures in both inguinal canals; the testicular parenchyma was markedly heterogeneous with nodular structures. We performed another pelvic MRI examination, which identified a residual testicular structure measuring 9.5 cm^3^ in the right hypogastrium. On diffusion weighted imaging (B = 600), the formation had high signal intensity and the apparent diffusion coefficient was >1; no paratesticular cysts were found. The lesions were characterized as benign; however, imaging could not rule out a premalignant transformation (Figure 1).

**Figure 2:**
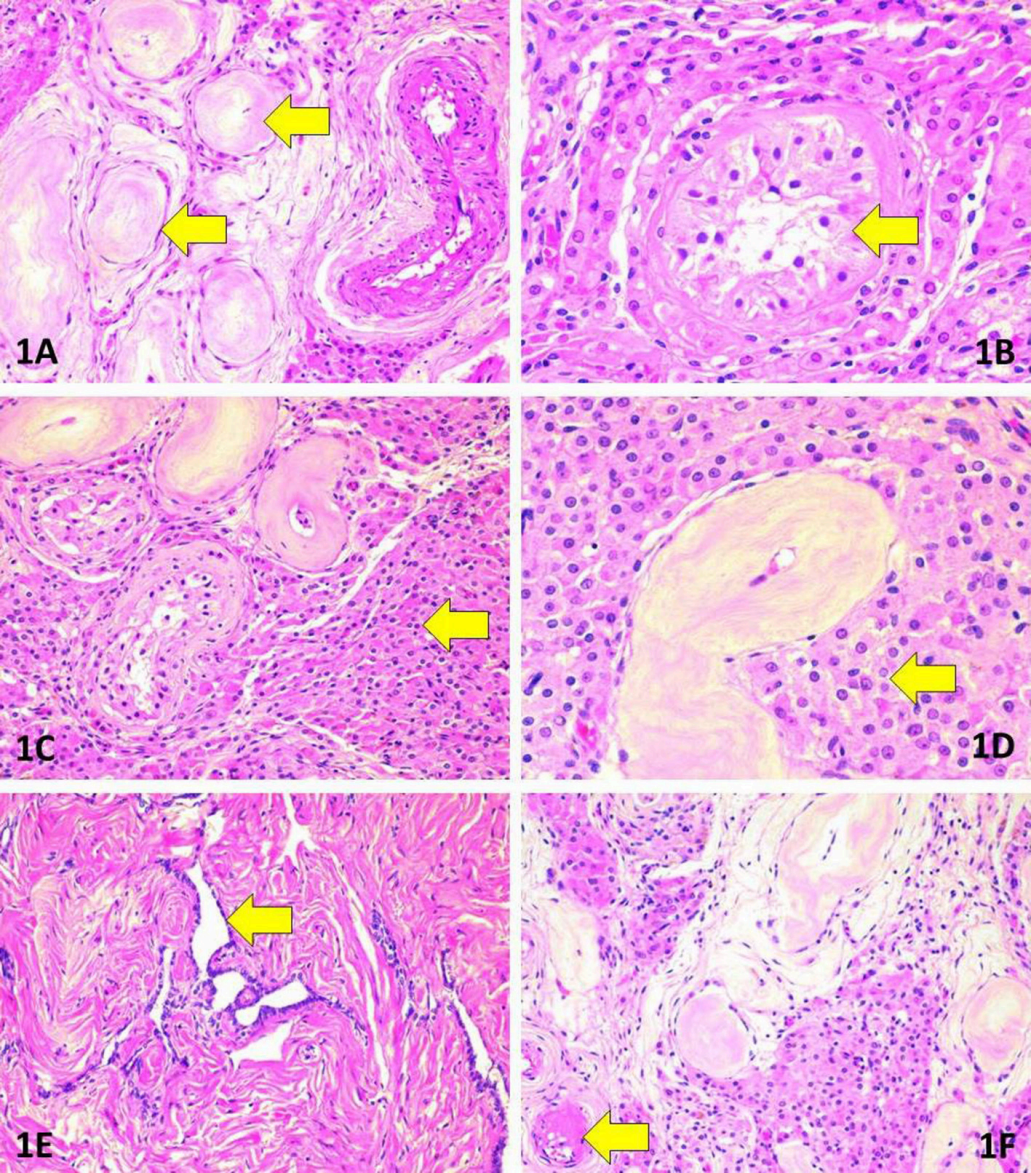
Morphologic findings in surgically removed testes. **(A)** Marked tubular atrophy. Note the thickening and hyalinisation of the basement membrane as well as complete loss of tubular cells (arrows). Haematoxylin-eosin (HE), original magnification (OM) 200x. **(B)** Absence of spermatogenesis (arrow). Note also the nuclear features justifying the absence of germ cell neoplasia *in situ*. HE, OM 400x. **(C)** Extensive hyperplasia of Leydig cells (arrow) adjacent to atrophic tubules. HE, OM 200x. **(D)** Morphology of Leydig cells (arrow). Note the absence of Reinke's crystalloids. HE, OM 400x. **(E)** Rete testis. HE, OM 200x. **(F)** Hyalinisation in an arteriole (arrow). HE, OM 200x.

The patient underwent a two-stage surgical intervention with the first stage of bilateral orchidectomy performed through an inguinal approach in September 2016. After visualization with haematoxylin-eosin stain, the testicular morphology showed extensive atrophic changes in the seminiferous tubules in association with Leidig cell hyperplasia (Figure 2). The morphology was remarkably heterogeneous in regard to the degree of atrophy and extent of Leidig cell hyperplasia. Seminiferous tubules featured extensive thickening of basement membranes along with hyalinisation of collagen. Consequently, the epithelium of most tubules had totally or almost completely disappeared, and these tubules were converted into homogeneous eosinophilic nodules. In the remaining tubules, Sertoli cells were found. The tubular lumina were either small or entirely lost. No signs of spermatogenesis were evident in multiple sections. Leydig cells were abundant although they lacked Reinke's crystalloids.

To exclude germ cell neoplasia *in situ*, the following intratubular cellular features were sought for: crowding and/or overlapping of nuclei, manifestations of cellular atypia such as increased cell diameter or presence of giant cells; enlarged, angulated, hyper- or hypochromatic nuclei; coarse structure of chromatin; nuclear pleomorphism; increased nucleo-cytoplasmic ratio; enlarged nucleoli; presence of mitoses, or atypical mitoses and/or foci of necrosis and dystrophic calcifications (microliths). Acknowledging the characteristically patchy nature of germ cell neoplasia *in situ*, multiple sections (3 per cm of diameter) were evaluated in haematoxylin-eosin stain. The intratubular cells did not show any of the listed traits of germ cell neoplasia *in situ*.

Atrophic rete testis was identified lacking any signs of cellular proliferation. The blood vessels were mostly intact but focal hyalinisation was evident in small arteries and arterioles.

Notably, one of the two obtained specimens consisted of two coalesced testes.

During the second stage of the surgical procedure, a laparoscopic orchidectomy was performed. The following histopathological examination yielded morphological findings that were identical with the previously described. After the second surgery, the patient's clinical presentation improved significantly; the postoperative laboratory results (hormonal analyzes taken in next day after the surgery) are shown in Table 1. In addition, 1 month after surgery, she had successfully reduced her weight to a BMI of 29.3 kg/m^2^ and had a stable blood pressure 125/80 mmHg.

Further recommendations included hormone replacement therapy with estradiol as a transdermal spray (*Lenzetto*® 1.53 mg), vaginal cream also containing estradiol (*Linoladiol*® 0.01%), and antihypertensive therapy.

### Genetic Analysis

To confirm the diagnosis genetically, PCR and bidirectional Sanger sequencing for the coding regions and exon/intron boundaries of the *AR* gene (NCBI Gene ID: 367, OMIM# 313700) were performed using a BigDye Terminator kit v.3.1. (Applied Biosystems, USA), according to the manufacturer's protocol. Previously described primers (8) were used, and the obtained sequence was analyzed and compared with the reference sequence NG_009014.2.

**Figure 3:**
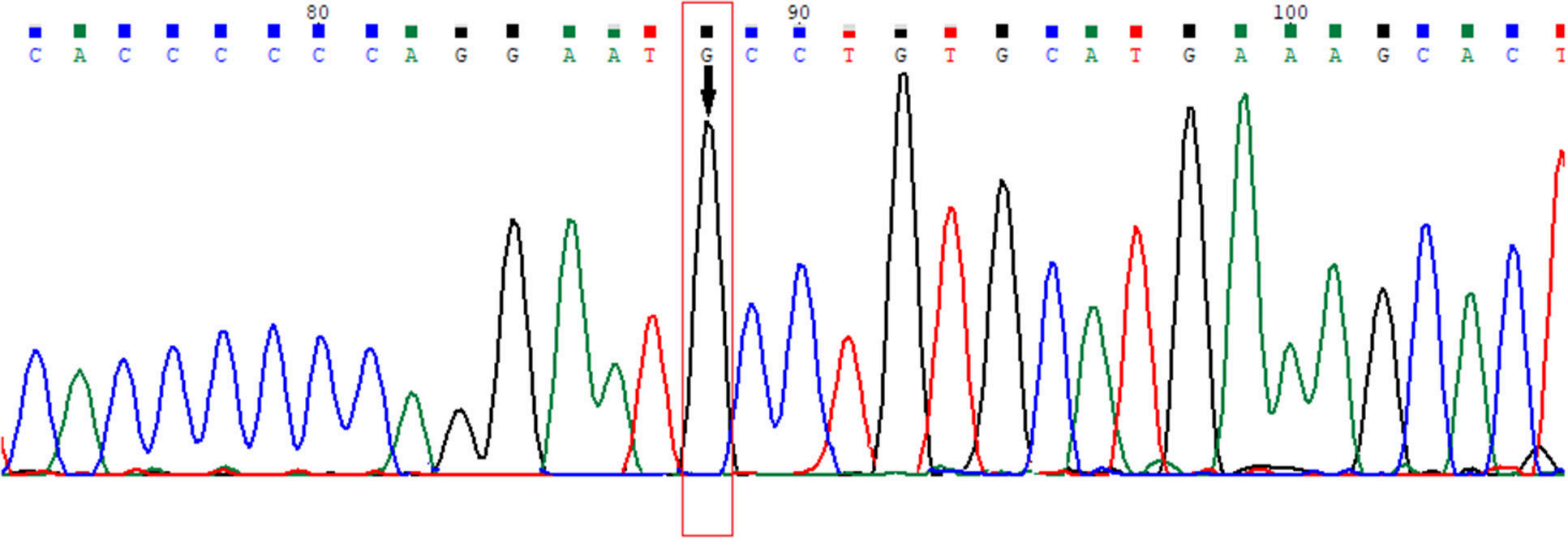
Electropherogram of the novel variation in the 6th exon of the *AR* gene (HGVS nomenclature: NC_000023.11:g.67721928T>G, NG_009014.2:g.182897T>G, NM_000044.3:c.2414T>G, NP_000035.2:p.Phe805Cys).

Previously unreported genetic variation with amino acid changes in the ligand binding domain (LBD) of the 6th exon of the *AR* gene, where phenylalanine at the 805th position was exchanged for cysteine (according to Human Genome Variation Society nomenclature: NC_000023.11:g.67721928T>G, NG_009014.2:g.182897T>G, NM_000044.3:c.2414T>G, NP_000035.2:p.Phe805Cys) was found (Figure 3). Guidelines for variation interpretation by the American College of Medical Genetics (ACMG) (9) were used to analyse the pathogenicity of the novel variation.

To analyse the frequency of the novel allelic variant, sequencing of 100 X chromosomes (25 women and 50 men) of the 6th exon of the *AR* gene was performed in healthy individuals from the general population of Latvia: none of them had the same variation, confirming that the detected variant was rare. There were no reported variations in this nucleotide according to the ClinVar database (10) at the time of preparation of this manuscript (06.07.2018.); however, two other variants in the same codon, where phenylalanine was exchanged for isoleucine and leucine (p.Phe805Leu and p.Phe805Ile) (11, 12) were reported in the Leiden Open Variation Database (LOVD) (13).

To computationally detect the pathogenicity of the identified variant, multiple *in silico* prediction tools as SIFT, PolyPhen, Condel, Mutation Taster were used (14–16), and it was classified as a deleterious variant (Pathogenity score in SIFT = 0; PolyPhen = 0.0997; Condel = 0.619; Mutation taster result—disease causing).

According to the ACMG variation interpretation guidelines, the discovered mutation was classified as likely pathogenic (PM2, PM5, PP3, PP4 criteria) confirming that CAIS could be diagnosed molecularly (9). Variant segregation in the patient' s family could not be performed because the patient did not have any relatives (the patient's parents were deceased, and the patient did not have any siblings).

## Discussion

This case report describes the characteristic findings of CAIS, such as primary amenorrhea, inguinal hernia (observed in adolescence), karyotype 46, XY and female phenotype. It is unique in the way notable distinctions were revealed in the form of long-term complications of retained testicular structures without malignancy despite improper repositioning, and in the rare form of polyorchidism or supernumerary testis (tetraorchidims) and novel genetic variant causing CAIS.

Polyorchidism is a very rare congenital anomaly. Only around 200 cases have been described in the literature until 2017: (17) and only several cases in last years reported tetraorchidism (18–21). To our knowledge, this is the first reported case of CAIS with polyorchidism although not the first in persons with disorders of sexual differentiations. The mechanism of polyorchidims is unknown, some researchers suggest it is caused of incomplete degeneration of the mesonephros or aberrant division or duplication of the genital ridge before 8 weeks gestation account for testicular duplications (21). Besides there are reports describing polyorhidism associated with chromosomal syndromes (17), and in number of cases in disorders of sexual differentiation (in karyotype 46,XX (22), 46,XY DSD (23), we believe that it is independent findings.

In patients with CAIS, it is recommended that gonadectomy be performed after puberty so that the occurrence of spontaneous puberty is not affected and because prepubertal malignancies are rarely reported (24). One of the largest studies performed involved the Cambridge Disorders of Sexual Development Database and evaluated 113 patients with CAIS, focusing on gonad malignancies. This study showed that the malignancy rate was 1.5%; however, only 13 patients underwent gonadectomy after 20 years of age; the others underwent surgery at an earlier age (25), so it was hard to evaluate long-term malignancies. However, in another study, it was reported that malignancies occur in up to 3.5% of all patients with CAIS who do not undergo gonadectomy by 25 years of age and in 33% of those who do not undergo gonadectomy by 50 years of age; it is also known that in patients with CAIS, malignancies are found more rarely than in patients with partial androgen insensitivity, confirming that the development of malignancy depends on residual androgen activity (26). The case described in this publication confirms that gonadectomy in patients with CAIS should be considered individually. Even though the malignancy rate is low at an earlier age, gonads can still affect the patient's clinical status later even without malignancy. The optimal follow-up strategy is also unclear because characteristic germ cell tumor markers, such as α-fetoprotein and β-HCG, are rarely elevated in association with seminomas and are never elevated in association with neoplasia *in situ* (27). Although targeted ultrasound or MRI can demonstrate the same suggestive findings even with early stage neoplasia, most often neoplasia remains undiagnosed with imaging methods (28, 29). The most available diagnostic technique is in theory is genetic variations (single nucleotide polymorphism) based malignancy risk susceptibility profiling (6). In our patient there were not observed any malignancy despite the fact that gonadectomy performed at age of 40ies.

The diagnosis of CAIS in the described patient was also confirmed with DNA analysis. A novel allelic variation was identified in the 6th exon of the *AR* gene that according to the ACMG interpretation guidelines (9) was classified as likely pathogenic thus confirming the disease. The frequency of detection of pathogenic variations in the *AR* gene in patients with CAIS ranges from 65 to 95% (30), although percentage could be even higher because in latest reports are described variants also in non-coding parts of the gene (31). The androgen receptor consists of three functional domains: an N-terminal transcription-regulation domain (encoded by the 1st exon), a DNA-binding domain (encoded by the 2nd and 3rd exons) and a C-terminal ligand-binding domain (LBD) (encoded by the 4–8th exons). In 56.5% of all patients with CAIS, including the patient described in this paper, allelic variation are located in LBD, thus this locations has the highest identified pathogenic variations density (32). Allelic variations in the LBD affect binding affinity or retention of dihydrotestosterone and testosterone to androgen receptors, thus leading to CAIS. Some *AR* LBD allelic variations do not affect ligand binding, but they disrupt androgen-induced N-terminal and C-terminal domain interaction, which also causes diminished androgen activity and results in a changed phenotype (CAIS) (1). We think that identified variant is causing CAIS but we don't have any evidence supporting hypothesis that it could be associated with polyorchidism.

The patient had a medical history included bilateral middle cerebral artery aneurysm that most likely is independent finding, however one similar case is described previously (33). Speculative correlation could be based on fact that patient had hypertension, that is known risk factor for cerebral aneurysm (34). However, male gender is protective as defective androgen receptor is defect abolishes sex differences in nitric oxide and reactivity to vasopressin so leading to more female gender specific effects (35).

Unfortunately, information regarding the patient's family was not available, so we could not determine whether the novel variant occurred *de novo* or was inherited; however in the first large cohort study by Morris (82 patients observed during the period from 1817 to 1953), it was reported that there was a strong familial connection, as shown by the number of sisters with the same findings (36); other studies also showed a high prevalence of inherited cases and mentioned *de novo* disease in only 30% of the patients (37). Patient infertility prevents direct inheritance from affected individuals.

## Data Availability Statement

All necessary information is shown in the manuscript.

## Author Contributions

IKo performed patient follow-up, clinical diagnosis, and manuscript preparation. JZ performed the molecular analysis, data interpretation, and manuscript preparation. AF collected the patient's clinical information and contributed to manuscript preparation. DR performed the genetic analysis. MD and IKa contributed to manuscript preparation. IS performed retrospective reevaluation for biopsies. LG checked the molecular data in the article and contributed to manuscript preparation.

### Conflict of Interest Statement

The authors declare that the research was conducted in the absence of any commercial or financial relationships that could be construed as a potential conflict of interest.

## References

[1] Jaaskelainen J Deeb A Schwabe JW Mongan NP Martin H Hughes IA. Human androgen receptor gene ligand-binding-domain mutations leading to disrupted interaction between the N- and C-terminal domains. J Mol Endocrinol. (2006) 36:361–8. 10.1677/jme.1.01885 16595706

[2] Hughes IA Davies JD Bunch TI Pasterski V Mastroyannopoulou K MacDougall J. Androgen insensitivity syndrome. Lancet (2012) 380:1419–28. 10.1016/S0140-6736(12)60071-3 22698698

[3] Gardner DG Shoback D Greenspan FS. Greenspan's Basic & Clinical Endocrinology. New York, NY: McGraw-Hill (2007).

[4] Hughes IA Houk C Ahmed SF Lee PA. Consensus statement on management of intersex disorders. archives of disease in childhood (2006) 91:554–63. 10.1136/adc.2006.098319 16624884 PMC2082839

[5] Cools M Drop SL Wolffenbuttel KP Oosterhuis JW Looijenga LH. Germ cell tumors in the intersex gonad: old paths, new directions, moving frontiers. Endocr Rev. (2006) 27:468–84. 10.1210/er.2006-0005 16735607

[6] Cools M Wolffenbuttel KP Hersmus R Mendonca BB Kaprova J Drop LSS. Malignant testicular germ cell tumors in postpubertal individuals with androgen insensitivity: prevalence, pathology and relevance of single nucleotide polymorphism-based susceptibility profiling. Hum Reprod. (2017) 32:2561–73. 10.1093/humrep/dex300 29121256

[7] Lee PA Nordenstrom A Houk CP Ahmed SF Auchus R Baratz A. Global disorders of sex development update since 2006: perceptions, approach and care. Horm Res Paediatr. (2016) 85:158–80. 10.1159/000442975 26820577

[8] Sharma V Thangaraj K Jyothy A. A novel insertion-induced frameshift mutation of the androgen receptor gene in a patient with primary amenorrhea. Meta Gene (2014) 2:11–5. 10.1016/j.mgene.2013.10.011 25606384 PMC4287795

[9] Richards S Aziz N Bale S Bick D Das S Gastier-Foster J. Standards and guidelines for the interpretation of sequence variants: a joint consensus recommendation of the American College of Medical Genetics and Genomics and the Association for Molecular Pathology. Genet Med. (2015) 17:405–24. 10.1038/gim.2015.30 25741868 PMC4544753

[10] Landrum MJ Lee JM Benson M Brown G Chao C Chitipiralla S. ClinVar: public archive of interpretations of clinically relevant variants. Nucleic Acids Res. (2016) 44:D862–8. 10.1093/nar/gkv1222 26582918 PMC4702865

[11] Gad YZ Mazen I Lumbroso S Temtamy SA Sultan C. A novel point mutation of the androgen receptor (F804L) in an Egyptian newborn with complete androgen insensitivity associated with congenital glaucoma and hypertrophic pyloric stenosis. Clin Genet. (2003) 63:59–63. 10.1034/j.1399-0004.2003.630109.x 12519373

[12] Cheikhelard A Morel Y Thibaud E Lortat-Jacob S Jaubert F Polak M. Long-term followup and comparison between genotype and phenotype in 29 cases of complete androgen insensitivity syndrome. J Urol. (2008) 180:1496–501. 10.1016/j.juro.2008.06.045 18710728

[13] Fokkema IF Taschner PE Schaafsma GC Celli J Laros JF den Dunnen JT. LOVD v.2.0: the next generation in gene variant databases. Hum Mutat. (2011) 32:557–63. 10.1002/humu.21438 21520333

[14] Kumar P Henikoff S Ng PC. Predicting the effects of coding non-synonymous variants on protein function using the SIFT algorithm. Nat Protoc. (2009) 4:1073–81. 10.1038/nprot.2009.86 19561590

[15] Gonzalez-Perez A Lopez-Bigas N. Improving the assessment of the outcome of nonsynonymous SNVs with a consensus deleteriousness score, Condel. Am J Hum Genet. (2011) 88:440–9. 10.1016/j.ajhg.2011.03.004 21457909 PMC3071923

[16] Adzhubei IA Schmidt S Peshkin L Ramensky VE Gerasimova A Bork P. A method and server for predicting damaging missense mutations. Nat Methods (2010) 7:248–9. 10.1038/nmeth0410-248 20354512 PMC2855889

[17] Uguz S Guragac A Demirer Z Yilmaz S Aydur E. Bilateral polyorchidism with ipsilateral two undescended testes: a rare congenital anomaly. Andrologia (2017) 49:e12643. 10.1111/and.12643 27373456

[18] Duymus M Menzilcioglu MS Cetincakmak M Avcu S. A rare case of polyorchidism: four testes. polish J Radiol. (2016) 81:39–41. 10.12659/PJR.895568 26893794 PMC4747319

[19] Alamsahebpour A Hidas G Kaplan A McAleer IM. Bilateral polyorchidism with diffuse microlithiasis: a case report of an adolescent with 4 testes. Urology (2013) 82:1421–3. 10.1016/j.urology.2013.06.039 24054439

[20] Repetto P Ceccarelli P Bianchini A Durante V Biondini D Cacciari A. Three small testes in left hemiscrotum: a rarer case of polyorchidism. J Pediat Surg. (2010) 45:e21–3. 10.1016/j.jpedsurg.2009.12.012 20152334

[21] Bergholz R Wenke K. Polyorchidism: a meta-analysis. J Urol. (2009) 182:2422–7. 10.1016/j.juro.2009.07.063 19765760

[22] Yoshida M Kakizawa Y Moriyama N Minowada S Higashihara E Aso Y. Deoxyribonucleic acid and cytological detection of Y-containing cells in an XX hypospadiac boy with polyorchidism. J Urol. (1991) 146:1356–8. 10.1016/S0022-5347(17)38093-X 1942291

[23] Renton CJ A case of polyorchidism with intersex. J Urol. (1975) 113:720–4. 10.1016/S0022-5347(17)59565-8 236401

[24] Mongan NP Tadokoro-Cuccaro R Bunch T Hughes IA. Androgen insensitivity syndrome. Best Pract Res Clin Endocrinol Metab. (2015) 29:569–80. 10.1016/j.beem.2015.04.005 26303084

[25] Chaudhry S Tadokoro-Cuccaro R Hannema SE Acerini CL Hughes IA. Frequency of gonadal tumours in complete androgen insensitivity syndrome (CAIS): a retrospective case-series analysis. J Pediat Urol. (2017) 13:498.e1–e6. 10.1016/j.jpurol.2017.02.013 28351649

[26] Kaprova-Pleskacova J Stoop H Bruggenwirth H Cools M Wolffenbuttel KP Drop SL. Complete androgen insensitivity syndrome: factors influencing gonadal histology including germ cell pathology. Mod Pathol. (2014) 27:721–30. 10.1038/modpathol.2013.193 24186138

[27] Schmoll HJ Souchon R Krege S Albers P Beyer J Kollmannsberger C. European consensus on diagnosis and treatment of germ cell cancer: a report of the European Germ Cell Cancer Consensus Group (EGCCCG). Ann Oncol. (2004) 15:1377–99. 10.1093/annonc/mdh301 15319245

[28] Nakhal RS Hall-Craggs M Freeman A Kirkham A Conway GS Arora R. Evaluation of retained testes in adolescent girls and women with complete androgen insensitivity syndrome. Radiology (2013) 268:153–60. 10.1148/radiol.13121068 23533290

[29] Hoei-Hansen CE Application of stem cell markers in search for neoplastic germ cells in dysgenetic gonads, extragonadal tumours, and in semen of infertile men. Cancer Treat Rev. (2008) 34:348–67. 10.1016/j.ctrv.2007.12.007 18289797

[30] Gottlieb B Beitel LK Nadarajah A Paliouras M Trifiro M. The androgen receptor gene mutations database: 2012 update. Hum Mutat. (2012) 33:887–94. 10.1002/humu.22046 22334387

[31] Kansakoski J Jaaskelainen J Jaaskelainen T Tommiska J Saarinen L Lehtonen R. Complete androgen insensitivity syndrome caused by a deep intronic pseudoexon-activating mutation in the androgen receptor gene. Sci Reports (2016) 6:32819. 10.1038/srep32819 27609317 PMC5016895

[32] Audi L Fernandez-Cancio M Carrascosa A Andaluz P Toran N Piro C. Novel (60%) and recurrent (40%) androgen receptor gene mutations in a series of 59 patients with a 46,XY disorder of sex development. J Clin Endocrinol Metab. (2010) 95:1876–88. 10.1210/jc.2009-2146 20150575

[33] Ju SM Yi HJ Ko Y Kim KM. Cerebral aneurysm and aortic coarctation in a 46, XY Female. Is it Causal or Coincidental? J Korean Neurosurg Soc (2005) 37:137–40. Available online at: www.jkns.or.kr/journal/view.php?number=2143

[34] Cebral JR Raschi M. Suggested connections between risk factors of intracranial aneurysms: a review. Ann Biomed Eng. (2013) 41:1366–83. 10.1007/s10439-012-0723-0 23242844 PMC3628920

[35] Stallone JN Salisbury RL Fulton CT. Androgen-receptor defect abolishes sex differences in nitric oxide and reactivity to vasopressin in rat aorta. J Appl Physiol. (2001) 91:2602–10. 10.1152/jappl.2001.91.6.2602 11717225

[36] Morris JM The syndrome of testicular feminization in male pseudohermaphrodites. Am J Obstet Gynecol. (1953) 65:1192–211. 10.1016/0002-9378(53)90359-7 13057950

[37] Hughes IA Deeb A. Androgen resistance. Best Pract Res Clin Endocrinol Metab. (2006) 20:577–98. 10.1016/j.beem.2006.11.003 17161333

